# The wide genetic landscape of clinical frontotemporal dementia: systematic combined sequencing of 121 consecutive subjects

**DOI:** 10.1038/gim.2017.102

**Published:** 2017-07-27

**Authors:** Cornelis Blauwendraat, Carlo Wilke, Javier Simón-Sánchez, Iris E Jansen, Anika Reifschneider, Anja Capell, Christian Haass, Melissa Castillo-Lizardo, Saskia Biskup, Walter Maetzler, Patrizia Rizzu, Peter Heutink, Matthis Synofzik

**Affiliations:** 1 Applied Genomics for Neurodegenerative Diseases, German Center for Neurodegenerative Diseases (DZNE), Tübingen, Germany; 2 Department of Neurodegenerative Diseases, Hertie Institute for Clinical Brain Research, University of Tübingen, Tübingen Germany; 3 Neurodegeneration, German Center for Neurodegenerative Diseases (DZNE), Tübingen, Germany; 4 Genome Biology of Neurodegenerative Diseases, German Center for Neurodegenerative Diseases (DZNE), Tübingen, Germany; 5 Biomedical Center (BMC), Biochemistry, Ludwig-Maximilians-Universität München, Munich, Germany; 6 Munich Cluster for Systems Neurology (SyNergy), Munich, Germany; 7 German Center for Neurodegenerative Diseases (DZNE) Munich, Munich, Germany; 8 Center for Genomics and Transcriptomics, Tübingen, Germany

**Keywords:** Alzheimer disease, cerebrotendinous xanthomatosis, frontotemporal dementia, neuronal ceroid lipofuscinosis, whole-exome sequencing

## Abstract

**Purpose:**

To define the genetic spectrum and relative gene frequencies underlying clinical frontotemporal dementia (FTD).

**Methods:**

We investigated the frequencies and mutations in neurodegenerative disease genes in 121 consecutive FTD subjects using an unbiased, combined sequencing approach, complemented by cerebrospinal fluid Aβ_1-42_ and serum progranulin measurements. Subjects were screened for *C9orf72* repeat expansions, *GRN* and *MAPT* mutations, and, if negative, mutations in other neurodegenerative disease genes, by whole-exome sequencing (WES) (*n* = 108), including WES-based copy-number variant (CNV) analysis.

**Results:**

Pathogenic and likely pathogenic mutations were identified in 19% of the subjects, including mutations in *C9orf72* (*n* = 8), *GRN* (*n* = 7, one 11-exon macro-deletion) and, more rarely, *CHCHD10*, *TARDBP, SQSTM1* and *UBQLN2* (each *n* = 1), but not in *MAPT* or *TBK1*. WES also unraveled pathogenic mutations in genes not commonly linked to FTD, including mutations in Alzheimer (*PSEN1*, *PSEN2*), lysosomal (*CTSF*, 7-exon macro-deletion) and cholesterol homeostasis pathways (*CYP27A1*).

**Conclusion:**

Our unbiased approach reveals a wide genetic spectrum underlying clinical FTD, including 11% of seemingly sporadic FTD. It unravels several mutations and CNVs in genes and pathways hitherto not linked to FTD. This suggests that clinical FTD might be the converging downstream result of a delicate susceptibility of frontotemporal brain networks to insults in various pathways.

## Introduction

The term “frontotemporal dementia” (FTD) describes a clinically and genetically heterogeneous group of disorders sharing progressive degeneration of frontotemporal networks as a common hallmark. Clinically, FTD comprises behavioral variant FTD (bvFTD), the semantic variant (svPPA), and the nonfluent variant (nfPPA) of primary progressive aphasia.^1, 2, 3^ However, behavioral and aphasic presentations of frontotemporal network degeneration can also be caused by underlying amyloid pathology, e.g., in behavioral variant AD (bvAD)^4^ and logopenic variant PPA (lvPPA).^3^ This is in line with several postmortem studies demonstrating that clinically and neuropathologically diagnosed FTD can result from different underlying pathologies (e.g., tau, TDP-43, or amyloid pathology), indicating that multiple pathogenic pathways might result in converging and/or overlapping clinical phenotypes.^5^

Corresponding with this complex pathological architecture of FTD, genes in manifold pathways have recently been implicated in its molecular pathogenesis.^6^ However, the full spectrum of neurodegenerative disease (NDD) genes and the relative proportions in which each contributes to the complex genetic architecture of FTD have not yet been systematically explored in a strictly consecutive series and using unbiased sequencing approaches. Here, we provide a systematic analysis of the frequencies and mutations in genes known to contribute to FTD/ALS and other NDDs in a consecutive series of 121 subjects with clinical FTD syndromes, combining successive Sanger sequencing, *C9orf72* repeat primed polymerase chain reaction (PCR), whole-exome sequencing (WES) and WES-based copy-number variation (CNV) analysis. We hypothesized that mutations are found in a substantial proportion of clinical FTD subjects, even in the absence of a positive family history, and also in genes hitherto not linked to FTD phenotypes.

## Materials and methods

### Subjects, clinical phenotyping, and biomarker analysis

A strictly consecutive series of 121 unrelated FTD subjects of Caucasian ancestry (over 90% from Southern Germany) was recruited at the Department for Neurodegenerative Diseases, Center for Neurology, Tübingen, Germany, from 2009 to 2014. All subjects were clinically diagnosed with FTD according to international consensus criteria.^1, 2^ Family history was positive for NDD in 33.9% (*n* = 41), as defined by the presence of one or more first- or second-degree relatives affected by any type of NDD. This study was approved by the Ethics Committee Tübingen, and written consent was obtained from all participants and their legal representatives.

All subjects received a systematic neurological assessment and, when possible, routine brain magnetic resonance imaging (MRI) (for further methods on clinical phenotyping, see Supplementary Material S1 online, for summary of the clinical features on the group level, see Table 1; for details of each subject, see Supplementary Material S2). Cerebrospinal fluid (CSF) amyloid-beta-42 (Aβ_1-42_), and serum progranulin levels were determined using commercially available ELISA sets for all individuals when CSF and serum, respectively, were available (CSF Aβ_1-42_ available for 97/121; serum progranulin available for 45/121) (for further methods, see Supplementary Material S1). This allowed investigation of the biomarker changes associated with both mutation-positive and mutation-negative clinical FTD.

### Genetic screening strategy and methods

A two-step genetic screening strategy was geared toward identifying a causal variant in as many subjects as possible, starting with the presumably common genetic causes in step 1, and continuing to rarer genetic causes in step 2 (Supplementary Material S3). In step 1, all subjects were tested for the presumably most common genetic causes of FTD in central Europe at the time of sequencing (2014), namely for repeat expansions in *C9orf72* (repeat lengths ≥30 considered pathogenic) as well as for variants in *GRN* and *MAPT.* The two latter genes were sequenced either selectively by Sanger sequencing or as part of a high-coverage high-throughput Agilent (Santa Clara, CA) SureSelect panel including 23 FTD, amyotrophic lateral sclerosis (ALS), and selected other early-onset dementia genes (for a complete list of the genes see Supplementary Material S4; for details on the genetic panel method, see Supplementary Material S1).

In step 2, all 108 subjects that were tested negative for repeat expansions in *C9orf72* and for pathogenic mutations in *GRN* and *MAPT* (or any other gene covered by the panel) were investigated by WES (Supplementary Material S4). Genes of interest were selected based on previous NDD gene searching^7^ and additional systematic literature review in search of genes implicated in FTD/ALS and other types of dementia. In total, 94 genes previously associated with FTD/ALS and other dementias (the set created based on existing literature in December 2015) were selected for analysis (for a full list of genes, see Supplementary Material S4). Quality control, gene-based and impact-score annotations, and population database frequencies were assigned to the variants, using ANNOVAR. Variants were filtered for (i) nonsynonymous coding variants (missense and loss of function (LOF) variants) and variants overlapping with putative splice sites (up to 25 bp of exon–intron junctions), that were (ii) absent or extremely rare (minor allele frequency <0.0005) in the public databases ExAC (version 0.3.1) and ESP6500 (http://exac.broadinstitute.org/, http://evs.gs.washington.edu/EVS/), and (iii) predicted pathogenic by at least one of the following in silico software algorithms: SIFT (J. Craig Venter Institute, La Jolla, CA), Polyphen-2, LRT, CADD (University of Washington and HudsonAlpha Institute for Biotechnology, Huntsville, AL) (Phred score ≥20) and DANN (Donald Bren School of Information and Computer Sciences, University of California, Irvine, CA) (score ≥0.98). The pathogenicity of the resulting variants was determined via a conservative multistep case-by-case analysis using the following criteria: (i) bioinformatic results on disease allele frequency and in silico predictions (same tools as for filtering), (ii) existing literature on these variants, (iii) manual curation on mutation type, domain location, and frequency in public databases, (iv) segregation analysis (where available), and (v) functional biomarkers known to be altered for pathogenic variants in the respective genes (e.g., progranulin for *GRN*, Aβ_1-42_ for *PSEN,* cholesterol metabolites for *CYP27A1*, arylsulfatase A activity for *ARSA* variants).

WES-based CNV analysis was applied using eXome-Hidden Markov Model (XHMM) software^8^ (for further details, see Supplementary Material S1). Positively curated CNVs were validated using quantitative PCR (qPCR) or multiplex ligation-dependent probe amplification. In total 112 subjects were investigated by at least one next-generation sequencing technique (panel: *n* = 33; WES: *n* = 108; *n* = 29 by both), thus also allowing simultaneous identification of possible multiple variants in several genes of interest in the majority of the included subjects.

## Results

### Overview of the identified genetic variants

**Figure 1:**
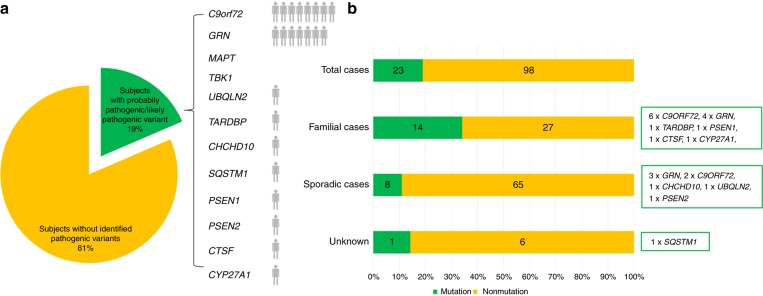
**Relative frequencies of mutations in neurodegenerative disease (NDD) genes in a consecutive series of 121 subjects with clinical frontotemporal dementia (FTD)**. Twenty-three subjects carried mutations, which were distributed across common FTD genes (*C9orf72* repeat expansion*, GRN*, but surprisingly not *MAPT* or *TBK1*), less common FTD genes (*CHCHD10*, *SQSTM1*, *TARDBP*, *UBQLN2*), and also NDD genes not commonly linked to FTD (*PSEN1*, *PSEN2*, *CTSF*, *CYP27A1*) (**a**). Mutations were found not only in 34% of familial subjects, but also in 11% of sporadic subjects (**b**).

Our combined sequencing approach yielded a total of 87 variants in the 94 analyzed FTD/ALS and other dementia genes, identified in 66 different individuals (for an overview of these 87 variants, see Supplementary Material S5). Twenty-four of the 87 variants, identified in 23 different individuals and affecting 10 distinct genes, were considered pathogenic or likely pathogenic (14 single nucleotide variants, eight *C9orf72* repeat expansions and two CNVs, one subject carrying two compound heterozygous variants) (Table 2 and Figure 1a), with nine variants being novel, i.e., not previously associated with human disease (for an overview, see Supplementary Material S5). This gives a total frequency of 23/121 (19%) mutation carriers with pathogenic or likely pathogenic variants. Family history in pathogenic/likely pathogenic mutation carriers was positive for NDD in 61% (14/23) subjects, while it was sporadic or unknown in 39% (9/23) of subjects. Pathogenic or likely pathogenic mutations (for the criteria, see “Materials and Methods”) were found in 11% (8/73) of sporadic subjects, encompassing largely the same gene spectrum as observed in the familial subjects (Figure 1b). Age of onset was significantly lower in the mutation carrier group than in the non-mutation carrier group (56.4 (SD = 11.5) vs. 64.2 (SD = 9.8) years, Mann-Whitney U test, two-tailed *P* < 0.0078).

### Pathogenic/likely pathogenic variants in established FTD genes

**Figure 2:**
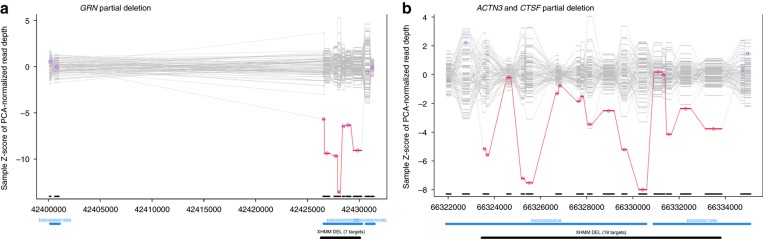
**Copy-number variants in** ***GRN*****and** ***CTSF*** **detected by whole-exome sequencing.** Two deletions were identified, affecting exons 2–12 of *GRN* in subject 18167 (**a**) and exons 1–6 of *CTSF* (plus exons 8–21 of *ACTN3*) in subject 19566 (**b**), respectively. The start and end points of both deletions were located in regions captured by the exome and the exact start and end points could be determined by visualizing the sequence data in the integrative genomics viewer (*GRN* deletion chr17:42,426,438–42,430,018; *CTSF* deletion chr11:66,323,324–66,333,606 (hg19)).

The most frequent finding was *C9orf72* repeat expansions, observed in eight subjects (8/121 (6.6%)), followed by pathogenic *GRN* variants in seven subjects (7/121 (5.7%)). Six of these seven *GRN* subjects carried truncating *GRN* variants (two frameshifts (c.759_760del:p.C253fs and c.985_986insAC:p.D329fs), two stop mutations (c.C328T:p.R110X and c.T687G:p.Y229X), one splicing variant (c.708+1G>A), and even one macro-deletion, spanning exon 2–13 (3.5 kb) (Figure 2a, confirmed by multiplex ligation-dependent probe amplification)). For three of the six subjects with truncating variants (one frameshift, one stop, and the large deletion), CSF and serum progranulin levels were available, all showing severely reduced progranulin levels (median CSF progranulin level: 1.8 ng/ml, median serum progranulin level: 10.4 ng/ml), for individual levels, see Supplementary Material S2), corroborating their pathogenicity. In addition to these six pathogenic LOF *GRN* variants, we identified one missense variant in *GRN* (c.C1117T:p.P373S, in subject 21862). For the following reasons, this variant might be pathogenic: it segregates with disease, is absent in ExAC, is predicted to be damaging by all five algorithms, affects an amino acid highly conserved through evolution, and is associated with reduced CSF progranulin levels, decreased to the same range as in LOF mutation carriers (CSF progranulin level in subject 21862: 2.4 ng/ml), thus indicating progranulin haploinsufficiency in the central nervous system compartment (for a detailed discussion of these findings, see Wilke et al.^9^). In contrast to previous reports on genetic FTD, we did not identify any pathogenic or likely pathogenic variants in *MAPT* or *TBK1*, despite good average coverage with >35x of the exonic regions.

In addition to variants in these presumed common FTD genes, we also identified four likely pathogenic variants in four less common FTD genes: *UBQLN2* (c.C845T:p.A282V), *TARDBP* (c.G1144A:p.A382T), *SQSTM1* (c.C1174G:p.P392A), and *CHCHD10* (c.C176T:p.S59L). As the *UBQLN2* and *TARDBP* variants have been described in detail elsewhere^10, 11^ (for functional proof of pathogenicity of the *TARDPB* A382T see Mutihac et al.^12^ Orru et al.^13^) we focus here on the *SQSTM1* and *CHCHD10* variants. Both variants are likely pathogenic for the following reasons: they are absent in ExAC, predicted to be damaging by all five algorithms, change an amino acid highly conserved through evolution, and affect the very same amino acid as do other already established pathogenic *SQSTM1* and *CHCHD10* mutations.

The *SQSTM1* variant affects the same amino acid as does another well-established, frequent *SQSTM1* mutation, namely the p.P392L mutation.^14^ The p.P392L mutation has almost exclusively been associated with FTD phenotypes complicated by either ALS^14^ or Paget disease of bone,^15^ or with ALS phenotypes, presenting the most common *SQSTM1* variant in ALS subjects from the United Kingdom.^16^ In contrast, the subject identified here (22531) presented with a pure bvFTD phenotype without signs of either ALS or parkinsonism, demonstrating that also *pure* FTD phenotypes can be part of the ALS-FTD phenotype spectrum caused by p.P392 *SQSTM1* amino acid changes. This subject showed no clinical signs of bone disease.

**Figure 3:**
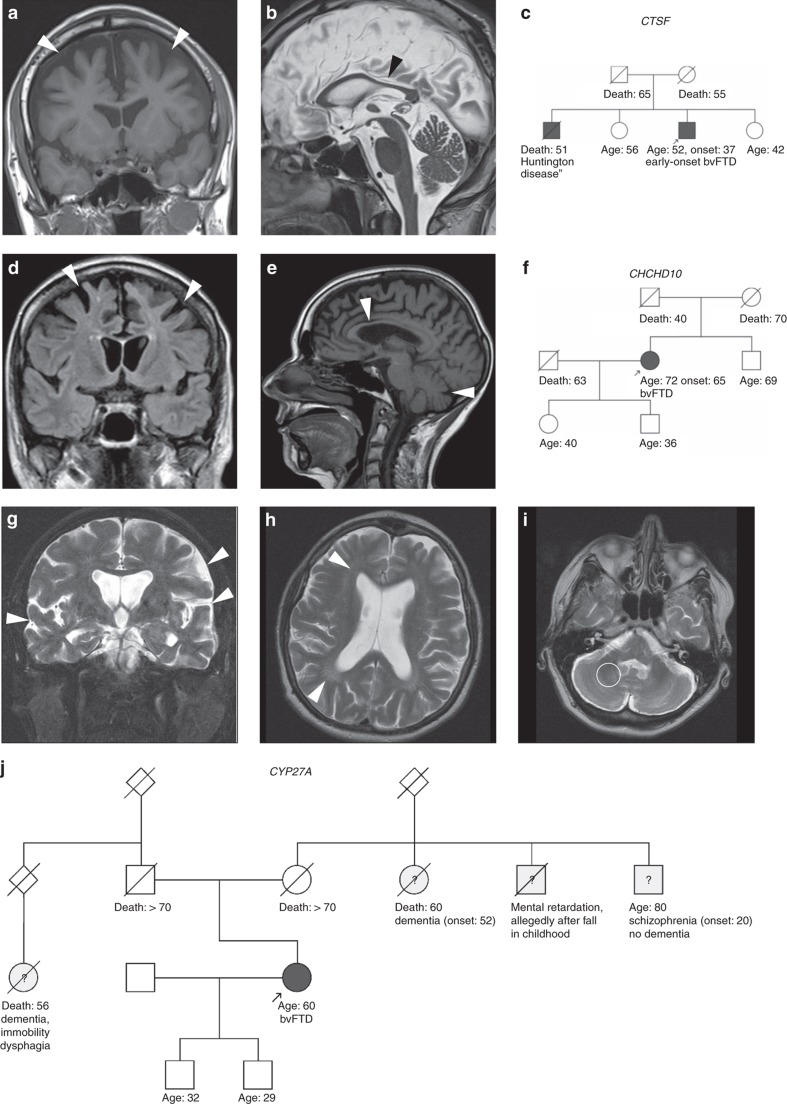
**Examples of rare genetic causes of clinical frontotemporal dementia (FTD): brain imaging and pedigrees of** ***CTSF***,***CHCHD10*** **and** ***CYP27A1*** **mutation carriers**. (**a**–**c**) *CTSF* subject. MRI of the *CTSF* subject (19566) showed frontotemporal atrophy (**a**) and thinning of the corpus callosum (**b**), but no definite white matter hyperintensity, demonstrating that *CTSF* mutations can present even with only unspecific FTD brain imaging changes. Family history revealed adult-onset behavioral change and cognitive decline in the deceased brother who was diagnosed with “Huntington disease” (**c**). (**d**–**f**) *CHCHD10* subject. MRI of the *CHCHD10* subject (21854) revealed bilateral frontal atrophy (**d**), mild cerebellar atrophy (**e**), and thinning of the corpus callosum (**e**). This subject appeared to be sporadic, but family history was incomplete owing to early death of the father (**f**). (**g**–**j**) *CYP27A1* subject. The MRI of the *CYP27A1* subject (23660) also showed predominantly temporal and frontal atrophy (**g**), and only unspecific, mild periventricular white matter changes (**h**), but no characteristic signal alterations of the dentate nucleus (**i**). This demonstrates that *CYP27A* mutations can also present with only unspecific FTD brain imaging changes, and can thus easily be overlooked in clinical practice. Clinical workup was at first misdirected by the presumed autosomal–dominant pattern of inheritance of a neuropsychiatric disease (**j**), before next-generation sequencing unraveled clearly pathogenic autosomal–recessive *CYP27A1* mutations in the index subject, indicating that there must be other causes for the neuropsychiatric diseases in the other family members (for a more detailed discussion of the subject’s family history, see Supplementary Material S7).

The p.S59L *CHCHD10* variant has been associated with a variety of syndromes, including motor neuron disease and/or (unspecified) dementia plus, in some subjects, cerebellar ataxia.^17^ Here we identified this variant in a bvFTD subject (21854) without signs of ALS or other NDD, thus reporting the first *pure* FTD phenotype of the p.S59L *CHCHD10* variant, and demonstrating that insults in mitochondrial proteins can lead to pure FTD (for MRI and pedigree, see Figure 3d–f; for a more detailed subject description, see Supplementary Material S6).

### Pathogenic and likely pathogenic variants in genes not commonly associated with clinical FTD

While variants in *PSEN1* and *PSEN2* have been linked to clinical FTD phenotypes before, reports on this association are still rare.^18^ In the present study we identified one pathogenic *PSEN1* splicing variant (c.869-2A>G; subject 19495) and one likely pathogenic *PSEN2* missense variant (c.T713C:p.L238P; subject 18506). In addition to the in silico data (see Supplementary Material S5), the pathogenicity of both *PSEN* variants was further corroborated by the finding of reduced CSF Aβ_1-42_ in both subjects (19495: 199 pg/ml; 18506: 440 pg/ml; for an earlier, detailed discussion of these findings, see Blauwendraat et al.^19^).

Surprisingly, we also observed one homozygous variant in *CYP27A1* (c.C1183A:p.R395S). This established mutation has been demonstrated to lead to alternative pre-mRNA splicing and decreased sterol 27-hydroxylase activity, thereby causing cerebrotendinous xanthomatosis.^20^ To confirm the pathogenicity of this variant, we confirmed the characteristic reduction of 27-OH-cholesterol (below the detection threshold), a sterol 27-hydroxylase product, and compensatory increases of 7-alpha-OH-cholesterol (1372 ng/ml) and cholestanol (3410 ng/dl) in our patient. This subject presented with impulsivity, disinhibition, apathy, and executive deficits at the age of 49 years, associated with pyramidal signs (for pedigree see Figure 3j; for more subject and pedigree details, see Supplementary Material S7). Remarkably, routine MRI revealed unspecific frontotemporal atrophy, but no specific cerebrotendinous xanthomatosis imaging changes (Figure 3g–i), demonstrating that this treatable condition can easily be overlooked in unexplained FTD subjects, even if caused by clearly pathogenic *CYP27A1* mutations.

In one subject (19566) we also found compound heterozygous mutations in the recently identified lysosomal lipofuscinosis gene *CTSF*.^21^ These include a missense variant (c.T1394G:p.L465W) located in *trans* with the first ever reported macro-deletion in this gene (10kb deletion spanning exons 6–13) (Figure 2b, deletion confirmed by multiplex ligation-dependent probe amplification). The missense variant is likely pathogenic as it is absent in ExAC, is predicted to be damaging by four of the five algorithms, and affects an amino acid highly conserved through evolution. This is the first report of a clinical FTD phenotype caused by *CTSF* mutations. The subject presented at the age of 37 years with an early-onset bvFTD phenotype comprising executive deficits, apathy, reduced empathy, and mild disinhibition, as well as clinical evidence of pyramidal involvement and mild apraxia. In contrast to previously described *CTSF* mutation carriers, the disease in this subject was not complicated by epileptic seizures, neither prior to dementia onset nor during the further course of the disease. Routine MRI revealed unspecific frontotemporal atrophy, but no specific imaging changes, demonstrating also that this condition can easily be overlooked in clinical practice (for MRI and pedigree, see Figure 3a–c; for a detailed subject description, see Supplementary Material S8). For more details of the identified variants, see Table 2 and Supplementary Material S5.

### Variants of uncertain significance, and deconstructing pathogenicity of the T410I ARSA variant

In addition, the WES filter settings (see “Materials and Methods”) yielded 63 variants of uncertain significance (VUS), which might be pathogenic, but for which strict evidence is currently lacking to classify them as potentially causative, according to our conservative variant interpretation approach. The observed VUS include changes in the genes *APP*, *ATXN2*, *CCNF*, *PRPH (*duplication), and *TBK1* (for a more detailed genetic and clinical discussion of the variants in these genes, see Supplementary Material S9).

The need for such a conservative, cautious approach when interpreting VUS in NDD genes is exemplified by the observed missense *ARSA* variant (c.C1229T:p.T410I). This variant, identified here in a homozygous state, is currently assumed to be pathogenic, reportedly causing “metachromatic leukodystrophy (MLD) presenting with a late-onset neuropathy,”^22^ and listed as pathogenic in ClinVar (http://www.ncbi.nlm.nih.gov/clinvar/variation/3092/). However, the subject identified here (20103) (i) presented with a clear bvFTD phenotype with age at onset of 65 years, which would be untypical for *ARSA*-associated disease/MLD; (ii) repeated detailed MRI at 67 and 71 years did not reveal any evidence for even subtle MLD changes; and, most importantly; (iii) repeated testing of arylsulfatase A activity was normal (1.43 IU/10^6^ cells, norm: >0.4 IU/10^6^ cells) (for a more detailed description, see Supplementary Material S10). These three findings provide evidence that the claim of pathogenicity for the p.T410I *ARSA* variant needs to be revised.

### Cerebrospinal fluid and serum biomarker findings

We observed a CSF Aβ42 reduction <550 pg/ml^23^ not only in the two *PSEN* subjects, but also in two individuals with clearly pathogenic *GRN* mutations (13413 and 18167) (Supplementary Material S11). Similarly, a serum progranulin reduction below the established <110 ng/ml threshold for *GRN* LOF mutations^24, 25^ was found not only in all the *GRN* LOF carriers for whom progranulin measurements were available but also in the *CHCHD10* missense carrier (Supplementary Material S11). These findings indicate that alterations in Aβ42 and progranulin levels are not restricted to AD and FTD-*GRN* subjects, respectively, but are a recurrent finding in other FTD subjects, representing possible downstream effects of mutations in other FTD genes (here: *GRN*/*CHCHD10*) and/or concomitant age-related amyloid/progranulin pathology.

## Discussion

### The genetic spectrum of clinical FTD: frequencies in a strictly consecutive series

Our study presents a systematic genetic in-depth study of a strictly *consecutive* series of clinical FTD subjects. Such a consecutive series, in which all subjects are either finally genetically solved or at least characterized as *C9orf72* repeat- and WES-negative, including WES-based CNV analysis, is crucial for unraveling the frequencies and distributions of genetic defects in clinical FTD. Other current frequency studies usually exclude subjects in whom a genetic defect has been found before (e.g., *C9orf72* expansions, *GRN*, or *MAPT*), describe nonconsecutive series based on prior explicit or implicit selection criteria, or study only single genes or a small set of genes, without screening *all* genes associated with neurodegenerative dementias, and thus do not provide an unbiased, representative estimate of the full genetic landscape of clinical FTD.^15, 26, 27^

In total, we identified 24 different pathogenic or likely pathogenic mutations in 23 subjects (23/121 (19%)), including eight seemingly sporadic subjects, and damaging ten different genes. This demonstrates that an unbiased genetic sequencing approach might allow the explanation of a substantial proportion of clinical FTD subjects, even in the absence of a positive family history. These numbers probably represent an underestimate, given that we identified 63 additional potentially pathogenic variants in FTD/ALS and other dementia genes and pathways, for which we took a conservative approach by classifying them as VUS. The negative family history in the mutation carriers with sporadic FTD (8/73 (11%) of all sporadic patients) might be explained by death of the parental generations before onset of disease; by underappreciated NDD in previous generations; and in particular by the incomplete penetrance which has been reported for almost all FTD genes. The fact that 9/23 (39%) of the subjects who had a positive family history for NDD did not show an obvious causal mutation demonstrates a still substantial amount of “missing heritability” in FTD genetics. This indicates the need for more advanced genetic techniques (e.g., whole-genome sequencing combined with RNA sequencing) allowing the capture of genetic variation in regulatory regions, RNA genes, and noncoding intronic regions.

### Frequencies of “standard” FTD genes

While the frequency of *C9orf72* repeat expansions (6.6%) and of *GRN* variants (5.8%) is in concordance with the reported European frequencies,^28, 29^ indicating that our subject cohort was broadly comparable to other FTD screening cohorts, we did not detect any probable pathogenic variants in *MAPT* and *TBK1*. Pan-European frequencies are reported at about 8% for *MAPT*^29^ and 0.4% for *TBK1*,^30^ with frequencies up to 17.8% for *MAPT*^31^ and 1.7% for *TBK1*^32^ in Dutch and Belgian populations, respectively. These findings add further evidence for substantial differences in mutation frequencies of common FTD genes, depending on the European population, with only very rare occurrences of *MAPT* mutations having been reported e.g., also in a Finnish cohort.^33^ These population differences have to be interpreted with caution, as they might also be partly influenced by between-center and between-country differences in subject recruitment. Nevertheless, they have to be accounted for when providing frequency numbers in the general public and for epidemiological studies, and when planning future observation and treatment studies in FTD.

While 15 of 121 (12.4%) subjects were thus explained by mutations in one of these assumed as common FTD genes that are frequently sequenced in clinical routine (*C9orf72*, *GRN*, *MAPT*, and *TBK1*), 8 (6.6%) of the FTD subjects could be explained by mutations in other genes. This has important implications for clinical practice and genetic diagnostics. It shows that manifold genetic causes of FTD are missed if only the FTD genes assumed as frequent are tested in the routine workup. Our approach thus exemplifies the power of unbiased next-generation sequencing, capturing *all* standard, non-standard FTD genes, including CNV analyses, and the need to introduce it into clinical practice. This will allow appreciating the extensive genetic background of clinical FTD syndromes.

### The wide genetic spectrum of clinical FTD: novel “nonstandard” FTD genes

Our unbiased genetic approach also allowed us to substantially extend the genetic spectrum underlying clinical FTD, unraveling several genes that have traditionally not been considered as FTD genes. For example, as shown here and elsewhere,^18^ mutations in *PSEN1* and *PSEN2* can present with a behavioral FTD syndrome, in which the clinical syndromes of bvFTD and behavioral variant AD^4^ become almost clinically indistinguishable (for more extensive discussions of these two mutation carriers see Blauwendraat et al.^19^). However, we now show that nonstandard dementia genes and models of autosomal-recessive inheritance also need to be considered in the workup and pathogenesis of clinical FTD syndromes. Our finding of a homozygous *CYP27A1* subject adds to the increasing list of unusual adult-onset cerebrotendinous xanthomatosis phenotypes^34^ and more specifically, adds support for FTD as a recurrent phenotype, as hypothesized by several single-case reports.^35, 36^

*CTSF* mutations have recently been identified as causing type B Kufs disease, an adult-onset neuronal ceroid lipofuscinosis, associated with a severe, early-onset neuropsychiatric phenotype with early epileptic seizures,^21, 37^ and more recently one family with early-onset AD phenotype has been reported.^38^ We now show that an adult-onset FTD phenotype, without seizures, can also be caused by *CTSF* mutations, thus extending the genetic and molecular basis of clinical FTD. This finding adds to the increasingly appreciated notion of insults in lysosomal pathways as an important contributor to the molecular architecture of FTD.^39^ Homozygous *GRN* mutations have already been shown to cause adult-onset neuronal ceroid lipofuscinosis^39^ and a pathobiochemical overlap of FTD and neuronal ceroid lipofuscinosis has been shown for Grn(−/−) mice (as a model for progranulin pathology) and, vice versa, also for Ctsd(−/−) mice (as a model for ceroid lipofuscinosis pathology) with progranulin being involved in the regulation of different cathepsin proteins.^39^ Our findings also extend the mutational spectrum of *CTSF* disease, by unraveling the first macro-deletion in *CTSF*. This shows the need to complement future genetic routine diagnostics of *CTSF* with CNV analyses that would not be captured by routine Sanger sequencing or standard WES analysis. In both subjects (*CYP27A1*, *CTSF*) MRI showed only unspecific frontotemporal atrophy, but no disease-specific changes (Figure 3), indicating that these conditions might easily be overlooked in subjects with unexplained FTD.

At the same time, our findings also revise some of the reported extended genotype–phenotype relations. The p.T410I variant in the MLD gene *ARSA* has been reported and to cause a mild, very late-onset neuropathy phenotype.^22^ We here report the first subject with this variant in a homozygous state, showing neither neuropathy nor MLD, and in particular exhibiting normal *ARSA* activity, thus casting doubt on the pathogenicity of this variant and thus of the reported *ARSA* phenotype. This finding illustrates the importance of constant critical clinical reanalysis of the manifold genetic variants produced by WES, which needs to include (as shown here) also variants previously reported to be pathogenic.

### Clinical FTD: a converging downstream result of manifold pathways?

Our findings of both classic and nonstandard dementia genes as causing clinical FTD syndromes also provide important insights into the molecular pathophysiology of FTD. They indicate that genetic insults contributing to this pathophysiology occur not only in pathways commonly linked to FTD (e.g., TDP-43, progranulin), but also in a wide variety of other pathways, e.g., mitochondrial (*CHCHD10*), amyloid (*PSEN1*, *PSEN2*), lipofuscinosis (*CTSF*), and cholesterol homeostasis (*CYP27A1*) pathways. This suggests that clinical FTD might be the converging downstream result of manifold pathways, arising from a delicate susceptibility of frontotemporal brain networks to genetic insults in these pathways. This notion is supported by recent gene coexpression network studies demonstrating that multiple disease mechanisms contribute to the pathology in the frontotemporal cortex in FTD subjects.^40^

Some of the defects in these pathways might be overlapping or converging when leading to the shared downstream clinical syndrome of FTD. Our findings provide preliminary indications that reductions in Aβ_1-42_ or progranulin might represent such partial overlaps or at least concomitant contributions to FTD disease etiology. Aβ_1-42_ reductions were observed not only in *PSEN* mutation carriers (where they would be expected), but also in *GRN* mutation carriers; and progranulin reductions were observed not only in *GRN* mutation carriers (where they are expected), but also in a *CHCHD10* mutation carrier. This notion of progranulin reduction as a shared feature across FTD subjects receives broader support from our recent finding that progranulin reductions are common even in FTD subjects without *GRN* mutations.^9^

In summary, we present here a genetic in-depth study of clinical FTD, unraveling mutations and CNVs in several genes hitherto not linked to FTD and, moreover, providing relative frequencies of a strictly *consecutive* series with clinical FTD. It demonstrates that genetic defects in various pathways contribute to the pathogenesis of clinical FTD, even including 11% of seemingly sporadic subjects. This suggests that comprehensive in-depth genetic screening might be considered in *all* FTD patients, even if family history is negative. Moreover, these findings indicate that clinical FTD is the converging downstream result of genetic insults in manifold pathways, arising from a delicate susceptibility of frontotemporal brain networks to insults in these pathways.

## Figures and Tables

**Table 1:** Clinical characteristics of the FTD cohort

**Total number of subjects**	121
Clinical syndrome	
bvFTD	58 (47.9%)
nfPPA	48 (39.7%)
svPPA	7 (5.8%)
lvPPA	8 (6.6%)
Additional features	
ALS	16 (13.2%)
Parkinsonism	12 (9.9%)
Family history of neurodegenerative disease	
Familial	41 (33.9%)
Sporadic	73 (60.3%)
Unknown	7 (5.8%)
Average age of onset	
All subjects	62.7 (range 30–84) years
Familial subjects	59.2 (range 30–81) years
Sporadic subjects	64.1 (range 32–83) years

ALS, amyotrophic lateral sclerosis; bvFTD, behavioral variant; lvPPA, logopenic variant primary progressive aphasia; nfPPA, nonfluent primary progressive aphasia; svPPA, semantic variant primary progressive dementia.

**Table 2:** Pathogenic and likely pathogenic variants identified in 121 clinical frontotemporal dementia subjects

**Gene, no. of mutation carriers (percentage) (*****n*** **= 121)**	**Subject**	**Phenotype**	**Variant**	**Amino acid change**	**Mutation class**	**MAF in ExAC**	**SIFT**	**Polyphen-2**	**LRT**	**CADD**	**DANN**
*C9orf72*, 8 (6.6%)	16265	nfPPA	Repeat expansion	n.a.	Repeat expansion	n.a.	n.a.	n.a.	n.a.	n.a.	n.a.
	18890	nfPPA									
	19115	nfPPA									
	19750	bvFTD									
	20879	bvFTD									
	21899	bvFTD									
	22181	nfPPA									
	29999	bvFTD									
*GRN*, 7 (5.8%)	13413	bvFTD	Exon7:c.T687G	Y229X	Stopgain	0	.	.	.	35	0.995
	18167	bvFTD	Exon 2–12 deletion	n.a.	Deletion (see Figure 2a)	.	.	.	.	.	.
	19869	nfPPA	Exon10:c.985_986insAC	p.D329fs	Frameshift insertion	0	.	.	.	.	.
	21804	bvFTD	Exon7:c.708+1G>A	n.a.	Splicing	8.28E−6	.	.	.	26.3	0.996
	21895	bvFTD	Exon10:c.C1117T	p.P373S	Missense	0	D	P	D	24.5	0.998
	23603	nfPPA	Exon4:c.C328T	p.R110X	Stopgain	0	.	.	.	29.4	0.994
	23812	svPPA	Exon8:c.759_760del	p.C253fs	Frameshift deletion	0	.	.	.	.	.
*UBQLN2*, 1 (0.8%)	18527	bvFTD	Exon1:c.C845T	p.A282V	Missense	0	D	D	D	24.7	0.999
*CHCHD10*, 1 (0.8%)	21854	bvFTD	Exon2:c.C176T	p.S59L	Missense	0	D	P	D	34	0.999
*SQSTM1*, 1 (0.8%)	22531	bvFTD	Exon8:c.C1174G	p.P392A	Missense	0	D	D	D	26.8	0.995
*TARDBP*, 1 (0.8%)	22458	bvFTD	Exon6:c.G1144A	p.A382T	Missense	0	T	B	N	13.06	0.98
*PSEN1*, 1 (0.8%)	18439	bvFTD	Exon9:c.869-2A>G	N/A	Splicing	0	.	.	.	24.9	0.995
*PSEN2*, 1 (0.8%)	18506	lvPPA	Exon8:c.T713C	p.L238P	Missense	0	D	D	D	26.8	0.999
*CYP27A1*, 1 (0.8%)	23660	bvFTD	Exon6:c.C1183A	p.R395S	Missense (homozygous)	8.25E−6	D	D	D	34	0.998
*CTSF*, 1 (0.8%)	19566	bvFTD	Exon13:c.T1394G	p.L465W	Missense +	0	D	D	D	27.1	0.966
	19566	bvFTD	Exons 1–6 deletion		Deletion (see Figure 2b)	.	.	.	.	.	.

B, benign; bvFTD, behavioral variant frontotemporal dementia; CADD, Combined Annotation Dependent Depletion; D, deleterious or probably damaging; DANN, deleterious annotation of genetic variants using neural networks; ExAC, Exome Aggregation Consortium database; LRT, likelihood ratio test; lvPPA, logopenic variant primary progressive aphasia; MAF, minor allele frequency; N, neutral; n.a, not applicable; nfPPA, nonfluent primary progressive aphasia; P, possibly damaging; SIFT, predicting the effects of coding non-synonymous variants on protein function using the SIFT algorithm; svPPA, semantic variant primary progressive aphasia; T, tolerant;

While the in silico predictions fail to predict pathogenicity in the TARDBP p. A382T variant, pathogenicity has been proven functionally by different groups and assays.^12, 13^
