# Supplementary material for: The wide genetic landscape of clinical frontotemporal dementia: systematic combined sequencing of 121 consecutive subjects
Source: Genet Med. 2017 Jul 27;20(2):240–9. doi: 10.1038/gim.2017.102 (PMC5846812; doi:10.1038/gim.2017.102)
Supplement: Supplementary Information [file gim2017102x1.docx]

**Supplementary Materials S1-11**

**The wide genetic landscape of clinical frontotemporal dementia: systematic combined sequencing of 121 consecutive subjects**

**Supplementary Material S1: Methodological details on clinical phenotyping, biomarker investigations, and genetic analyses**

***Clinical phenotyping.*** Concomitant amyotrophic lateral sclerosis (ALS) was diagnosed according to the revised El Escorial criteria ^1^. Parkinsonism was diagnosed if bradykinesia and at least one of the following was present: muscular rigidity, 4-6 Hz rest tremor or postural instability ^2^.

***Cerebrospinal fluid and serum biomarkers.*** Cerebrospinal fluid (CSF) amyloid-beta-42 (Aß_1-42_) and serum progranulin were assessed to explore the biomarker changes associated with both mutation-positive and mutation-negative clinical FTD. Aß_1-42_ and progranulin levels were determined using commercially available ELISA sets for all individuals where CSF and serum, respectively, were available (CSF Aß1-42 available for 97/121 and serum progranulin available for 45/121) (ELISA Aß_1-42_: Innotest β-amyloid ELISA by Fujirebio, Ghent, Belgium; ELISA progranulin: Adipogen AG, Liestal, Switzerland). Within our clinically defined cohort, we considered Aß_1-42_ levels < 550 pg/ml as indicative of parenchymal amyloid pathology ^3^, and serum progranulin levels < 110 ng/ml as indicative of progranulin insufficiency ^4,5^. We did not use the Aß_1-42_ threshold as an exclusion criterion for excluding subjects from our clinical FTD cohort, as this could result in excluding those FTD subjects who have amyloid pathology as downstream effects of FTD gene mutations and/or concomitant amyloid pathology.

***Panel sequencing*.** For panel sequencing, genomic DNA was enriched by a custom-made Agilent SureSelect in-solution kit, followed by next generation sequencing of these genes using a barcoded library on one full slide on the SOLiD 5500xl platform (Life Technologies) generating approximately 10 million mappable 75 bp reads. For previous descriptions of this panel method see ^6^.

***Whole exome sequencing analysis*.** WES libraries were prepared using Agilent Technologies SureSelect V5 and subjected to 100 or 125-base pair paired-end sequencing on an Illumina HiSeq2000, HiSeq2500 or HiSeq4000. Sequence reads were aligned to the reference genome (hg19) using the Burrows-Wheeler Aligner (BWA) mem algorithm of the BWA software package (version 0.7.9a) ([http://bio-bwa.sourceforge.net](http://bio-bwa.sourceforge.net/)). Picard tools (version 1.129) (<http://broadinstitute.github.io/picard/>) was used to create .bam files and to sort and index the sequence reads. Single nucleotide variants and small insertion/deletions were called, recalibrated, multi allelic variant split and left normalization using the Genome Analysis Toolkit (GATK, version 3.3-0) (<https://www.broadinstitute.org/gatk/>), following the recommended workflow for variant analysis.

***WES-based copy number variant analysis.*** Copy number variants (CNVs) were identified using eXome-Hidden Markov Model (XHMM) software, following the developer’s guidelines ^7^. In brief, depth of coverage statistics were calculated per sample of all genes of interest using GATK (version 3.3-0), then normalized using principal component analyses and filtered based on target size and target coverage. Common CNVs (MAF > 0.05) and CNVs located in high GC and low complexity regions were removed. Identified CNVs were plotted and visually inspected. Positively curated CNVs were validated using quantitative PCR (qPCR) or multiplex ligation-dependent probe amplification (MLPA).

**Supplementary Material S2:** **Table with subject characteristics**

Supplementary_Material_S1_cohort_FTD_exome.xlsx

**Supplementary Material S3: Strategy of genetic analysis.**


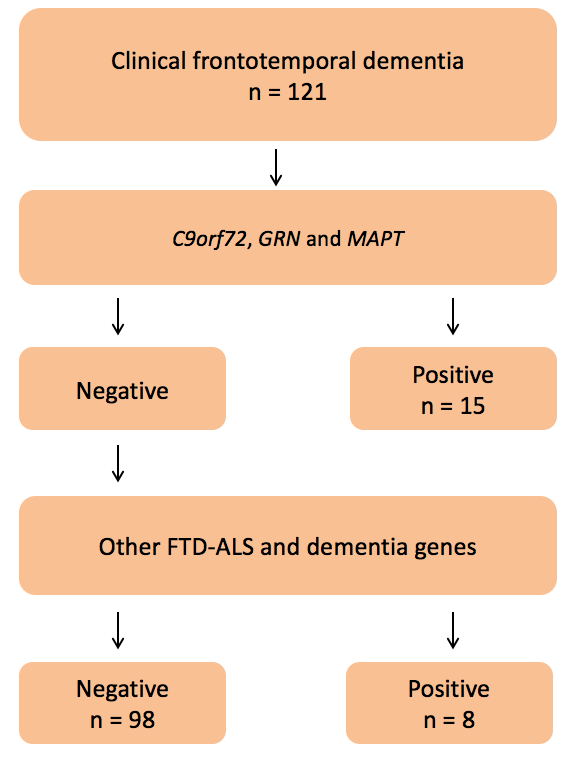


Figure: **Strategy of genetic analysis**. Step1: Subjects were screened for *C9orf72* repeat expansions, *GRN* and M*APT* mutations. Step 2: If negative, they were then also screened for mutations in other FTD-ALS and dementia genes by whole exome sequencing (WES), including WES-based copy number variant analysis.

**Supplementary Material S4:** **Table with screened genes by whole exome sequencing and targeted panel sequencing**

Supplementary_Material_S4_genelist_FTD_exome.xlsx

**Supplementary Material S5: Table with all potentially pathogenic variants**

Supplementary_Material_S5_variants_FTD_exome.xlsx

**Supplementary Material S6: Subject characteristics of subject #21854, *CHCHD10* p.S59L, heterozygous**

The subject presented at the age of 68 years with a three-year history of behavioural variant frontotemporal dementia (bvFTD), comprising of a progressive dysexecutive syndrome and personality change in the form of apathy, social withdrawal and reduced empathy (MMSE 15/30 points). Family history was negative for dementia, motor neuron disease and parkinsonism, but of limited informative value as the father had died early (Figure 3, main text). Clinical examination additionally revealed some semantic paraphasia, but provided no evidence of additional motor neuron disease, parkinsonism or cerebellar ataxia. It thus presents the first pure frontotemporal dementia (FTD) phenotype of the p.S59L *CHCHD10* variant, without signs of amyotrophic lateral sclerosis (ALS) or other neurodegenerative disease. MRI revealed bilateral frontal atrophy (Figures A, B, E, F) and, in addition, mild cerebellar atrophy (E, F) and thinning of the corpus callosum (D) without relevant white matter lesions (B, C). CSF analysis did not suggest parenchymal amyloid pathology (amyloid-beta-42 888 pg/ml, t tau 456 pg/ml, p-tau 51 pg/ml).


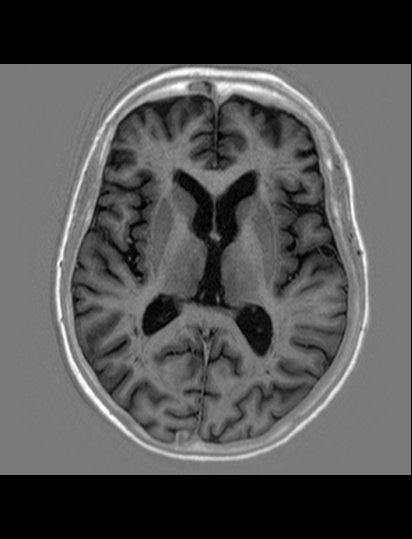


C


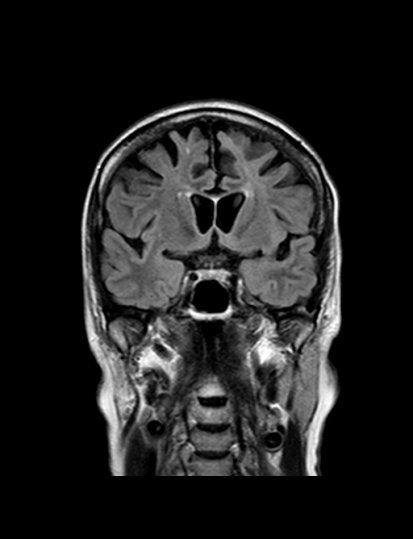


A


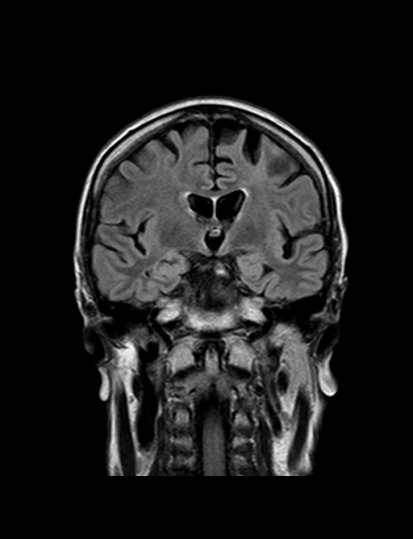


B


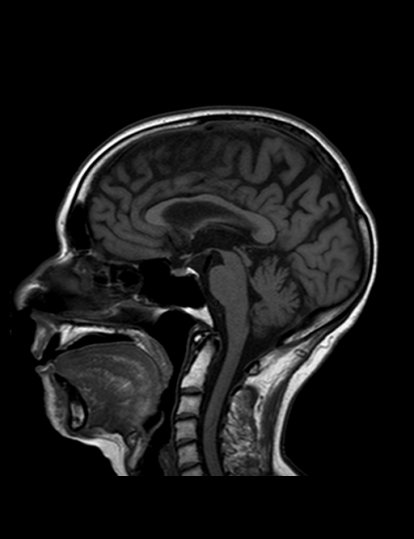


D


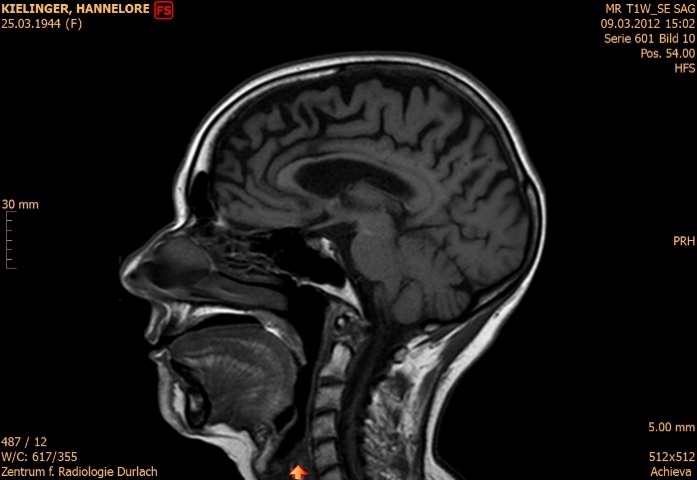


E


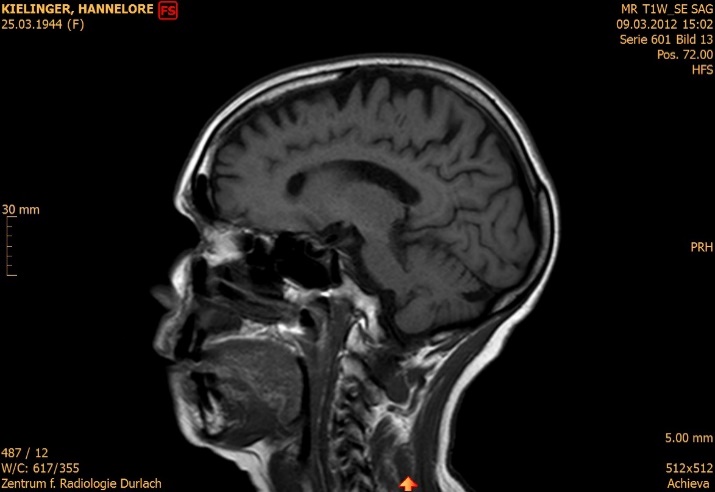


F

**Supplementary Material S7: Subject characteristics of subject #23660, *CYP27A1*; p.R395S, homozygous**

The subject presented with a progressive syndrome of impulsivity, disinhibition, apathy, executive deficits and clinical pyramidal signs at the age of 49 years, preceded by several years of depressed mood. Family history was initially mis-interpreted as autosomal-dominant neuropsychiatric disease (in particular dementia) with incomplete penetrance (see pedigree in Figure 3, main text). Clinical re-evaluation upon identification of the *CYP27A1* variant revealed surgery for bilateral cataracts (age: 40 years) and bilateral Achilles-tendon xanthomas (age: 30 years), compatible with clinical criteria of cerebrotendinous xanthomatosis (CTX). Laboratory testing confirmed reduction of 27-OH-cholesterol (below detection threshold), a sterol 27-hydroxylase product, and compensatory increases of 7-alpha-OH-cholesterol (1372 ng/ml) and cholestanol (3410 ng/dl). MRI revealed predominantly temporal and frontal atrophy and mild unspecific periventricular white matter changes, but no characteristic signal alterations of the dentate nucleus (see Figure 3, main text). Cognition declined further until treatment with chenodesoxycholic acid was started at age 60 years (MMSE: 23/30 (age 44 years), 20/30 (age 59 years), 18/30 (age 60 years), 21/30 (age 61 years)).

This subject finding demonstrates that a clinical FTD phenotype can be caused by *CYP27A1* mutations, and that, correspondingly, disturbances in cholesterol pathways can lead to degeneration of frontotemporal networks. Moreover, this subject illustrates that the interpretation of a family history need to be constantly scrutinised. FTD phenotypes in subjects with a seemingly autosomal-dominant family history might in fact be caused by autosomal-recessive mutations, as illustrated by this subject. In turn, as shown by the findings from our whole FTD cohort, FTD phenotypes in seemingly sporadic subjects might also be caused by autosomal-dominant mutations; see Figure 1B, main text. This illustrates the benefits of recent unbiased next-generation sequencing techniques in the work-up of FTD which allow to find the responsible gene even when a different pattern of inheritance (and thus a different gene set) had initially been conjectured. It is highly likely that the neuropsychiatric disease in the parental generations of the *CYP27A1* index subject results from other causes than biallelic *CYP27A1* mutations.

**Supplementary Material S8: Subject characteristics of subject #19566, *CTSF* deletion exons 6-13; c.1394 T>G, p.L465W, compound heterozygous**

A 37-year-old man presented with an early-onset bvFTD phenotype comprising of executive deficits, apathy, reduced empathy and mild disinhibition. Clinical examination indicated additional pyramidal signs and mild apraxia. Family history revealed adult-onset behavioural change and cognitive decline in the deceased brother (death with 51 years), diagnosed with “Huntington’s disease” (see pedigree Figure 3, main text). However, genetic testing of the index subject was negative for mutations in genes causing Huntington’s and Huntington-like diseases (*HTT*, *JPH3* and *TBP)*. MRI demonstrated frontotemporal atrophy (Figure A and B) and thinning of the corpus callosum (Figure C), but no definite white matter hyperintensity (Figure D). CSF analysis did not suggest parenchymal amyloid pathology (amyloid-beta-42 1479 pg/ml, t-tau 309 pg/ml, p-tau 57 pg/ml). The disease course showed marked cognitive decline (MMSE 25/30 with 46 years, MMSE 10/30 with 50 years), but epileptic seizures remained absent. This is the first report of a FTD phenotype caused by *CTSF* mutations, and the first report of a *CTSF* macro-deletion.


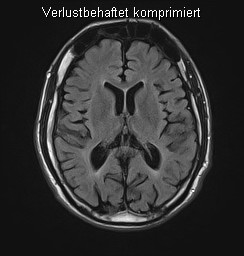


D


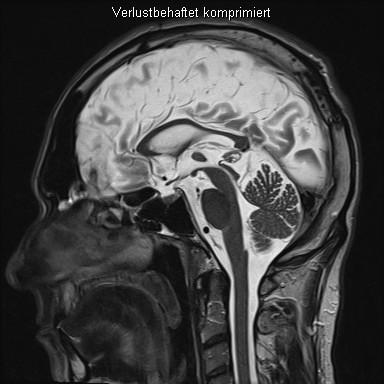


C


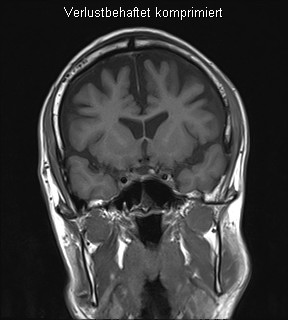


A


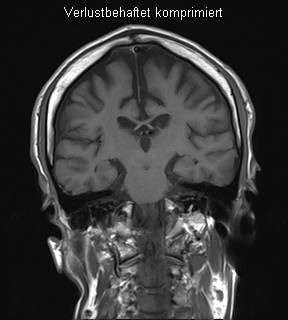


B

**Supplementary Material S9: Detailed clinical and genetic analysis of variants of unknown significance in *APP*, *ATXN2*, *CCNF*, *PRPH (*duplication) and *TBK1***

Our WES filter settings yielded 63 additional variants in the 94 genes investigated of unknown significance, which might be pathogenic, but for which strict evidence is currently lacking to classify them as potentially causative. This included variants in the genes *APP, ATXN2, CCNF, PRPH* (duplication) *and TBK1*. A detailed genetic and clinical discussion of the variants is provided here.

***APP.*** We identified a missense mutation in *APP* (c.G1995C:p.E665D, rs63750363) in a subject (#23923) presenting with a PNFA phenotype in combination with ALS. This variant has been previously found in a late onset AD subject, and CSF biomarker findings in the index subject were compatible with underlying amyloid pathology (Aß_1-42_ 478 pg/ml; t-tau 558pg/ml; p-tau 60pg/ml). However, this variant has previously been identified also in a non-affected family member ^8^, and the phenotype in the index subject was not typical for *APP*-associated disease (presence of ALS).

***ATXN2***. In subject #13208, we identified a splice variant at the beginning of exon 13 of *ATXN2* disrupting the acceptor sequence by changing from AG to AC (c.2237-1G>C). This variant is absent in ExAC, the splice sequence is conserved through evolution and has a high CADD score 26.6. CAG repeats in *ATXN2* have been shown to be a major cause of spinocerebellar ataxia 2 and additionally associations have been made between repeat length and ALS and progressive supranuclear palsy ^9^. While the proposed genetic mechanism of pathogenicity of *ATXN2* repeats is gain of function, deficiency of *ATXN2* has been suggested an important role in various neurodegenerative processes ^10,11^. It is thus tempting to speculate that also the LOF conferred by this splice site mutation might lead to disease. However, no tissue or cells were available from this subject to confirm this splice effect on exon 13.

***CCNF.*** Recently, variants in *CCNF* have been reported to cause ALS and/or FTD ^12^. We here identified a novel missense variant (c.C591A:p.F197L), located very close to a reported potentially pathogenic variant (p.S195R) identified in a Spanish familial ALS subject. However, this variant is also identified in four subjects from the ExAC database, therefore pathogenicity is less likely.

***PRPH.*** In subject #19203, we identified a duplication of the *PRPH* gene (see Figure below). To validate this *PRPH* copy number variant, we performed a copy number variation quantification experiment on DNA isolated from the index subject and four control samples using the Taqman copy number assay Hs01937474_cn (Applied Biosystems) for the *PRPH* gene and the RNAse P Taqman copy number reference assay (Applied Biosystems). Duplex real-time PCR reactions were run on a ViiA™ 7 Real-Time PCR System (Applied Biosystems) according to the manufacturer’s protocol. Results were analysed using the CopyCaller™ Software (Applied Biosystems). All four controls were having two copies of the *PRPH* gene and the index subject had three copies, confirming the heterozygote duplication.

The subject #19203 presented with a PNFA phenotype with parkinsonism, beginning at age 73 years. *PRPH* missense variants have previously been associated with ALS ^13,14^. Interestingly, overexpression of *PRPH* in mice results in an ALS phenotype ^15^. Given the large genetic overlap between ALS and FTD, this gives rise to the interesting hypothesis that this duplication could contribute to the disease phenotype. Unfortunately, however, tissue or cells were not available from this subject to confirm a potential increase in *PRPH* expression.


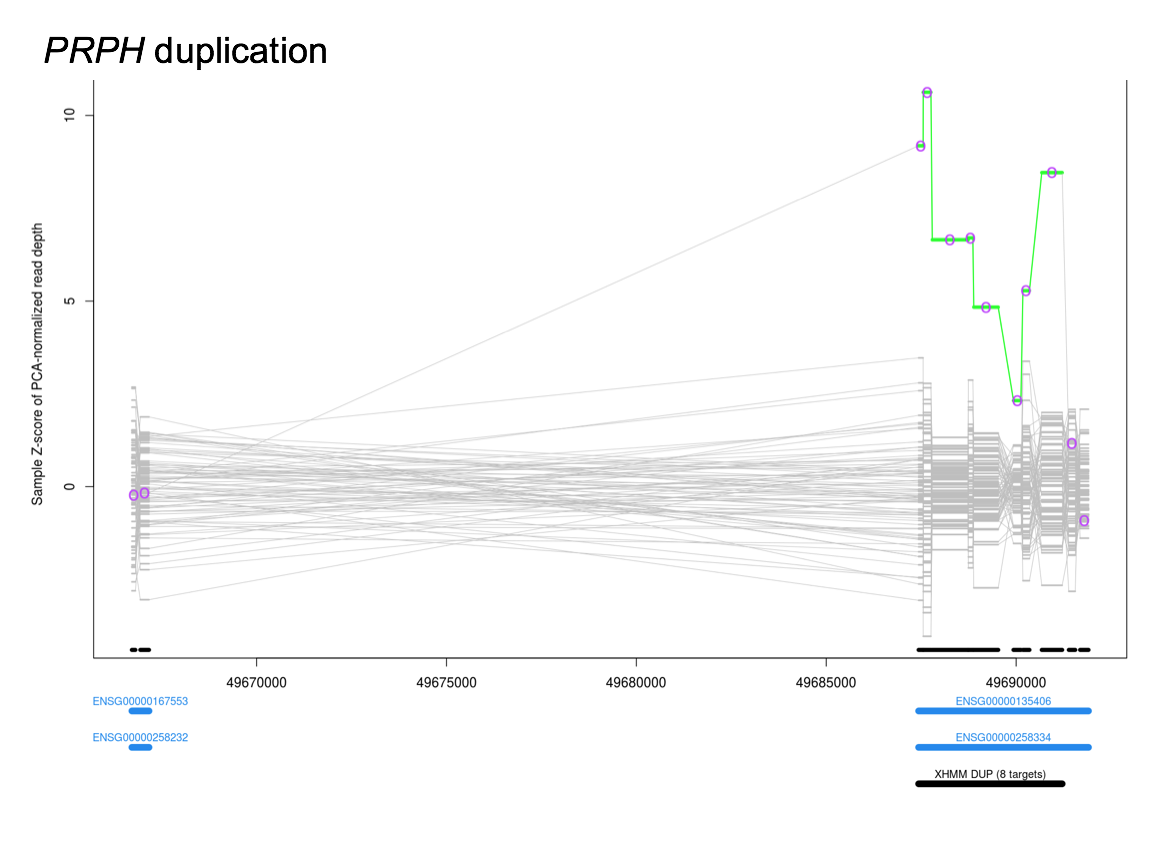


Figure: **Copy number variants in *PRPH* detected by whole exome sequencing.** The start and end point of the *PRHP* duplication of subject #19203 were likely outside the captured area and could not be determined.

***TBK1.*** We identified two missense mutations (c.A1445G:p.Y482C and c.T2063A:p.L688H) and one potential splicing variant (c.228+6T>C) in *TBK1*, all absent in ExAC. Missense and LOF mutations in *TBK1* have been reported to cause FTD/ALS ^16-18^. Pathogenicity is complicated to prove for missense mutations in *TBK1,* as disease is typically caused by haploinsufficiency through LOF mutations. Therefore, pathogenicity of the two missense mutations is unclear, but cannot be precluded.

The splicing variant (identified in **subject #** **17927)** was predicted to affect the splicing of exon 3, resulting in a shorter transcript missing exon 3. We aimed to confirm this splicing effect by non-quantitative RT-PCR. RNA was isolated from peripheral blood mononuclear cells with the RNeasy kit (Qiagen) including DNAse treatment. RNA integrity (RIN) was determined on a Tape Station 2200 system (Agilent Technologies Inc.). Total RNA primed with oligo dT (Qiagen) and random decamers (Thermo Fisher Scientific) was used for cDNA synthesis with Superscript III reverse transcriptase (RT) (Thermo Fisher Scientific) according to manufacturer’s specifications. Non-quantitative PCR on cDNAs from the index subject (#17927) and three control samples was carried out using the following pairs of primers (primer pair 1: forward 5'-actgcaaatgtctttcgtgga-3' and reverse 5'-acagtgtataaactcccacatgg-3'; primer pair 2: forward 5'- gcaaatgtctttcgtggaagac-3' and reverse 5'- caccacatctcgcaaaacaa-3'). On agarose gel we could observe two bands, a higher band corresponding to the full *TBK1* transcript and a faint lower band corresponding to the short transcript lacking exon 3. Thus, the shorter transcript missing exon 3 could be confirmed. In a next step, quantitative PCR was carried out in triplicate on a ViiA7 real time PCR system (Applied Biosystems) on cDNAs from the index subject and seven control samples using SYBR Green PCR master mix (Thermo Fisher Scientific) and 0,04 μM specific primer pair forward 5'-atttgctattgaagaggagacaac-3' and reverse 5'-cagtgtataaactcccacatgga-3'. Comparative Ct values (ΔΔCt values) were calculated using the real-time PCR system v1.2 (Applied Biosystems) with *TBP1*, *PPIA1*, *PPIB2* and *OAZ1* as reference targets. No differences were identified in *TBK1* exon 3 expression levels between the splicing variant carrier and the control.

**Supplementary Material S10: Subject characteristics of subject #20103, *ARSA* p.T410I homozygous**

The subject presented at the age of 67 years with a two-year history of bvFTD, comprising of apathy, reduced empathy, impulsivity and a dysexecutive syndrome. Family history over three generations was negative for dementia, motor neuron disease and parkinsonism (Figure A). Clinical examination additionally showed frontal signs, reduced spontaneous speech and anosognosia, but did not suggest any peripheral neuropathy, pyramidal tract involvement or basal ganglia involvement. MRI revealed temporal (B, E), hippocampal (C, F) and also frontal (E, F) atrophy, with clear progression of cerebral atrophy over time (B-D: 67 years, E-H: 71 years). However, MRI did not reveal any evidence for even subtle metachromatic leukodystrophy (MLD) changes (no leukoencephalopathy in D, G and H) and repeated testing of enzymatic *ARSA* activity was normal 1.43 IU / 10^6^ cells, norm: > 0.4 IU / 10^6^ cells). These findings revise the alleged pathogenicity of the p.T410I *ARSA* variant, which has been reported earlier ^19^.


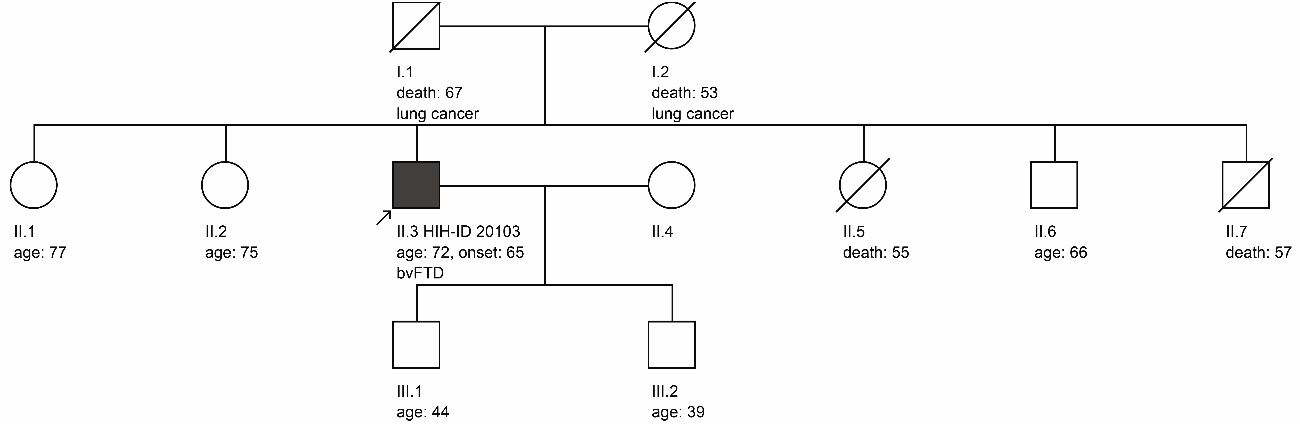


A


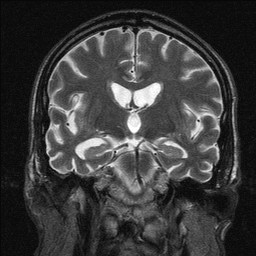


C


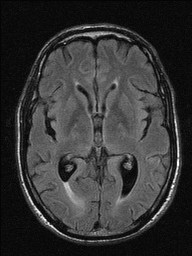


D


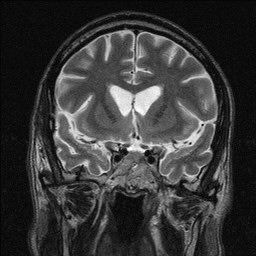


B


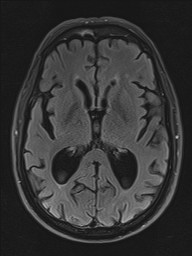


G


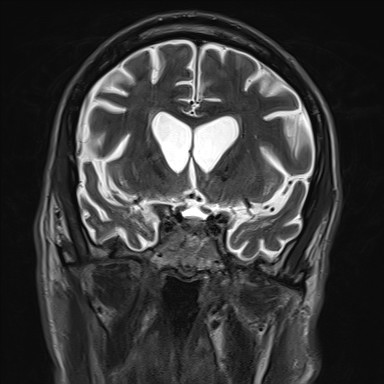


E


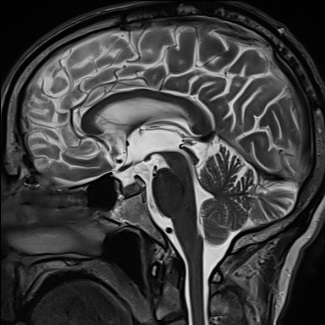


H


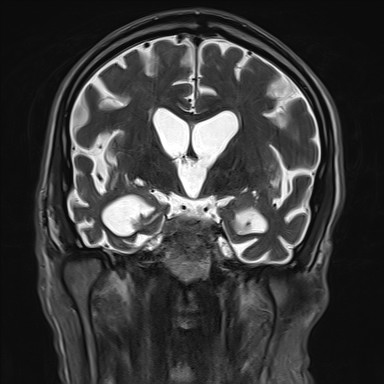


F

**Supplementary Material S11: Frequencies of reduced Aβ42 and progranulin levels in mutation and non-mutation carriers.** Bar graphs show the relative frequencies of CSF Aβ42 reductions (< 550 pg/ml) (A) and serum progranulin reductions (< 110 ng/ml) (B) of mutation carriers, non-mutation carriers and the entire cohort, respectively (red bars = number of subjects per group with reduced levels of Aß_1-42_ and progranulin, respectively; blue bars = number of subjects per group with normal levels of Aß_1-42_ and progranulin, respectively). Reduced CSF Aß_1-42_ was observed not only in two individuals with *PSEN* mutations, but also in two individuals with *GRN* mutations (A). Reduced serum progranulin was observed in the three subjects with *GRN* mutations of whom serum progranulin measurements were available, but also in the individual with the pathogenic *CHCHD10* variant (B), suggesting that alterations of progranulin levels might extend beyond *GRN* loss-of-function mutations. Absolute numbers of available measurements are indicated by numbers.


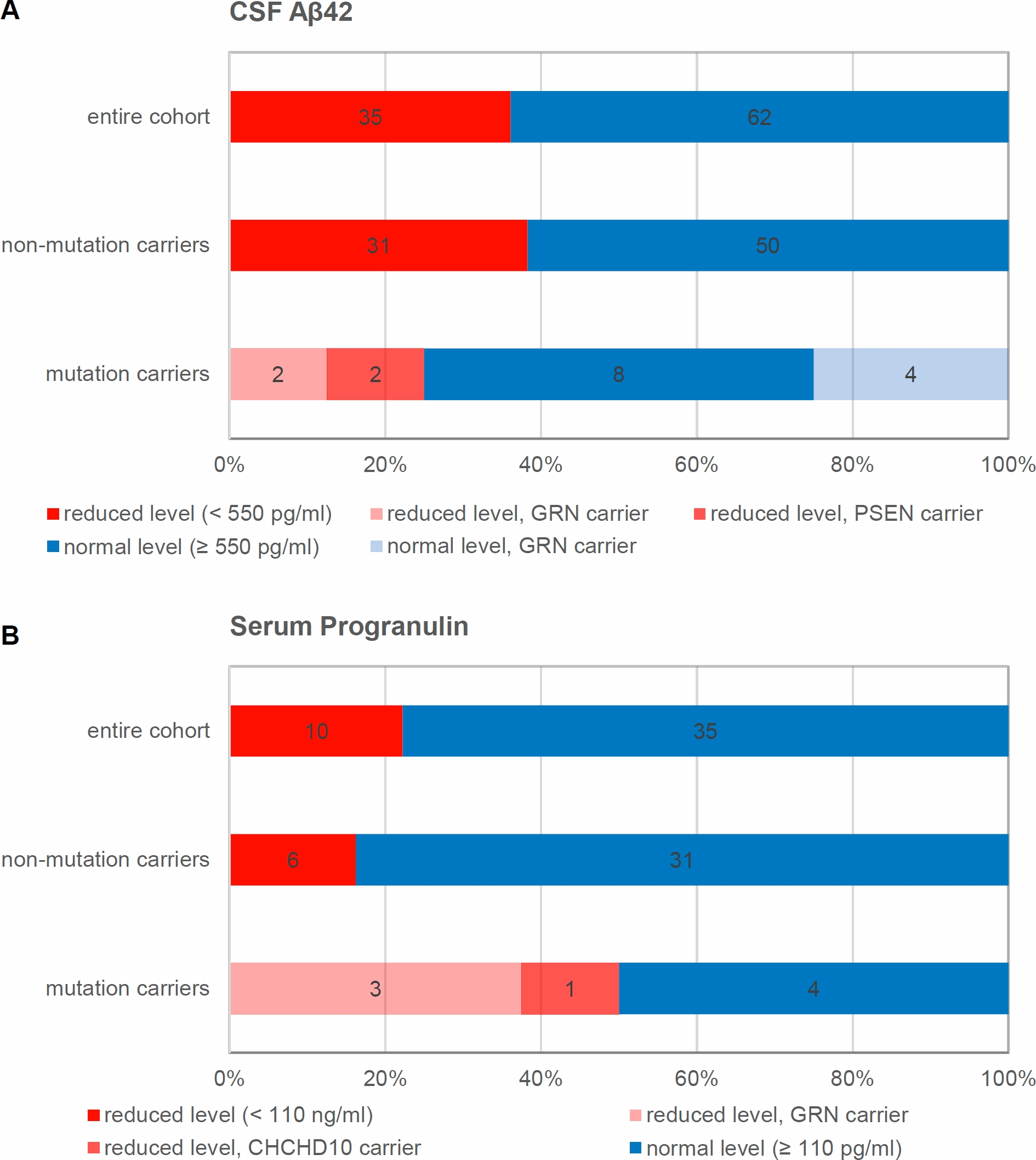


**References**

1. Brooks BR, Miller RG, Swash M, Munsat TL, World Federation of Neurology Research Group on Motor Neuron D. El Escorial revisited: revised criteria for the diagnosis of amyotrophic lateral sclerosis. *Amyotroph Lateral Scler Other Motor Neuron Disord.* 2000;1(5):293-299.

2. Hughes AJ, Daniel SE, Kilford L, Lees AJ. Accuracy of clinical diagnosis of idiopathic Parkinson's disease: a clinico-pathological study of 100 cases. *J Neurol Neurosurg Psychiatry.* 1992;55(3):181-184.

3. Mulder SD, van der Flier WM, Verheijen JH, et al. BACE1 activity in cerebrospinal fluid and its relation to markers of AD pathology. *J Alzheimers Dis.* 2010;20(1):253-260.

4. Finch N, Baker M, Crook R, et al. Plasma progranulin levels predict progranulin mutation status in frontotemporal dementia patients and asymptomatic family members. *Brain.* 2009;132(Pt 3):583-591.

5. Ghidoni R, Benussi L, Glionna M, Franzoni M, Binetti G. Low plasma progranulin levels predict progranulin mutations in frontotemporal lobar degeneration. *Neurology.* 2008;71(16):1235-1239.

6. Synofzik M, Born C, Rominger A, et al. Targeted high-throughput sequencing identifies a TARDBP mutation as a cause of early-onset FTD without motor neuron disease. *Neurobiol Aging.* 2014;35(5):1212 e1211-1215.

7. Fromer M, Purcell SM. Using XHMM Software to Detect Copy Number Variation in Whole-Exome Sequencing Data. *Curr Protoc Hum Genet.* 2014;81:7 23 21-21.

8. Peacock ML, Murman DL, Sima AA, Warren JT, Jr., Roses AD, Fink JK. Novel amyloid precursor protein gene mutation (codon 665Asp) in a patient with late-onset Alzheimer's disease. *Ann Neurol.* 1994;35(4):432-438.

9. Ross OA, Rutherford NJ, Baker M, et al. Ataxin-2 repeat-length variation and neurodegeneration. *Hum Mol Genet.* 2011;20(16):3207-3212.

10. Meierhofer D, Halbach M, Sen NE, Gispert S, Auburger G. Ataxin-2 (Atxn2)-Knock-Out Mice Show Branched Chain Amino Acids and Fatty Acids Pathway Alterations. *Mol Cell Proteomics.* 2016;15(5):1728-1739.

11. Al-Ramahi I, Perez AM, Lim J, et al. dAtaxin-2 mediates expanded Ataxin-1-induced neurodegeneration in a Drosophila model of SCA1. *PLoS Genet.* 2007;3(12):e234.

12. Williams KL, Topp S, Yang S, et al. CCNF mutations in amyotrophic lateral sclerosis and frontotemporal dementia. *Nat Commun.* 2016;7:11253.

13. Gros-Louis F, Lariviere R, Gowing G, et al. A frameshift deletion in peripherin gene associated with amyotrophic lateral sclerosis. *J Biol Chem.* 2004;279(44):45951-45956.

14. Leung CL, He CZ, Kaufmann P, et al. A pathogenic peripherin gene mutation in a patient with amyotrophic lateral sclerosis. *Brain Pathol.* 2004;14(3):290-296.

15. Beaulieu JM, Nguyen MD, Julien JP. Late onset of motor neurons in mice overexpressing wild-type peripherin. *J Cell Biol.* 1999;147(3):531-544.

16. Freischmidt A, Wieland T, Richter B, et al. Haploinsufficiency of TBK1 causes familial ALS and fronto-temporal dementia. *Nat Neurosci.* 2015;18(5):631-636.

17. Pottier C, Bieniek KF, Finch N, et al. Whole-genome sequencing reveals important role for TBK1 and OPTN mutations in frontotemporal lobar degeneration without motor neuron disease. *Acta Neuropathol.* 2015;130(1):77-92.

18. Gijselinck I, Van Mossevelde S, van der Zee J, et al. Loss of TBK1 is a frequent cause of frontotemporal dementia in a Belgian cohort. *Neurology.* 2015;85(24):2116-2125.

19. Comabella M, Waye JS, Raguer N, et al. Late-onset metachromatic leukodystrophy clinically presenting as isolated peripheral neuropathy: compound heterozygosity for the IVS2+1G-->A mutation and a newly identified missense mutation (Thr408Ile) in a Spanish family. *Ann Neurol.* 2001;50(1):108-112.
